# Experience in reverse sequence procedures for esophageal cancer surgery

**DOI:** 10.1186/1749-8090-10-S1-A41

**Published:** 2015-12-16

**Authors:** Hsu Chung-Ping, Chih-Hung Lin, Cheng-Yen Chuang

**Affiliations:** 1 Division of Thoracic Surgery, Department of Surgery, Taichung Veterans General Hospital, Taichung, Taiwan, ROC; 2 School of Medicine, National Yang-Ming University, Taipei, Taiwan, ROC

## Background/Introduction

Despite different surgical techniques, conventional approaches for esophageal cancer surgery comprise of tumor resection followed by esophageal reconstruction.

## Aims/Objectives

Been a high volume cancer center, we aim to investigate the efficacy and safety of reverse sequence procedures (reconstruction first followed by resection) in treating esophageal cancer patients.

## Method

Being a high volume cancer center, we aim to investigate the efficacy and safety of reverse sequence procedures (reconstruction first followed by resection) in treating esophageal cancer patients.

## Results

After excluding 13 conversions (5 in reverse group, 8 in non-reverse group), the operation time, blood loss, and retrieved lymph nodes number, cervical anastomotic leak, and hospital stay were 468.6 vs. 506.3 min (p = 0.004), 420.1 vs. 286.7 cc. (p = 0.012), 37.4 vs. 29.6 (p = 0.002), 20 vs. 15 cases (p = 0.008), and 14.4 vs. 17.0 days (p = 0.034), in reverse group and non-reverse group, respectively. There were 2 hospital mortalities, complete pathologic response was obtained in 44 of the 119 neoadjuvant patients (37.0%), and the cumulative 5-yr survival rates were 45.3%.

## Discussion/Conclusion

Reverse sequence MIE is an efficient and safe procedure in treatment of esophageal patient cancers, which also greatly facilitates the procedure of esophagectomy.

